# miR-1 as a tumor suppressive microRNA targeting TAGLN2 in head and neck squamous cell carcinoma

**DOI:** 10.18632/oncotarget.213

**Published:** 2011-01-25

**Authors:** Nijiro Nohata, Yaeko Sone, Toyoyuki Hanazawa, Miki Fuse, Naoko Kikkawa, Hirofumi Yoshino, Takeshi Chiyomaru, Kazumori Kawakami, Hideki Enokida, Masayuki Nakagawa, Makio Shozu, Yoshitaka Okamoto, Naohiko Seki

**Affiliations:** ^1^ Department of Functional Genomics, Chiba University Graduate School of Medicine, Chiba, Japan; ^2^ Department of Otorhinolaryngology/Head and Neck Surgery, Chiba University Graduate School of Medicine, Chiba, Japan; ^3^ Department of Gynecologic Oncology, Chiba University Graduate School of Medicine, Chiba, Japan; ^4^ Department of Urology, Graduate School of Medical and Dental Sciences, Kagoshima University, Kagoshima, Japan

**Keywords:** microRNA, miR-1, TAGLN2, tumor suppressor, HNSCC, microarray, oncogenes, oncotargets

## Abstract

Based on the microRNA (miRNA) expression signatures of hypopharyngeal and esophageal squamous cell carcinoma, we found that *miR-1* was significantly down-regulated in cancer cells. In this study, we investigated the functional significance of *miR-1* in head and neck squamous cell carcinoma (HNSCC) cells and identified *miR-1*-regulated novel cancer pathways. Gain-of-function studies using *miR-1* revealed significant decreases in HNSCC cell proliferation, invasion, and migration. In addition, the promotion of cell apoptosis and cell cycle arrest was demonstrated following *miR-1* transfection of cancer cells. A search for the targets of *miR-1* revealed that *transgelin 2* (*TAGLN2*) was directly regulated by *miR-1*. Silencing of *TAGLN2* significantly inhibited cell proliferation and invasion in HNSCC cells. Down-regulation of *miR-1* and up-regulation of *TAGLN2* were confirmed in HNSCC clinical specimens. Our data indicate that *TAGLN2* may have an oncogenic function and may be regulated by *miR-1*, a tumor suppressive miRNA in HNSCC. The identification of novel *miR-1*-regulated cancer pathways could provide new insights into potential molecular mechanisms of HNSCC carcinogenesis.

## INTRODUCTION

Head and neck squamous cell carcinoma (HNSCC) constitutes the sixth most common malignancy worldwide [1]. In spite of considerable advances in multimodality therapy including surgery, radiotherapy, and chemotherapy, the overall five year survival rate for patients with this type of cancer is among the lowest of all major cancer types and has not improved during the last decade [2]. Local tumor recurrence and distant metastasis after conventional therapy appear to be major contributing factors for restricted survival of HNSCC patients. Therefore, understanding the molecular oncogenic pathways underlying HNSCC would help to improve diagnosis, approaches to therapy, and prevention of the disease.

MicroRNAs (miRNAs) are endogenous, short, non-coding RNA molecules which regulate gene expression by translational repression or degradation of mRNA in a sequence-specific manner [3]. Bioinformatic prediction indicates that miRNAs regulate more than 30% of the protein coding genes [4]. It is estimated that approximately 1,000 miRNAs exist in the vertebrate genome. At this time, 1,048 human miRNAs are registered at miRBase release 16.0 (http://microrna.sanger.ac.uk/). A growing body of evidence suggests that miRNAs are aberrantly expressed in many human cancers, and that they play significant roles in carcinogenesis and cancer progression [5]. miRNAs can be divided into two classes: those which are oncogenic miRNAs and those which are tumor suppressive miRNAs. Up-regulated miRNAs could act as oncogenes by negatively regulating tumor suppressor genes, while down-regulated miRNAs could function as tumor suppressors by repressing oncogenes [6,7].

Studies of tumor suppressive miRNAs and searches for their target genes are important for our understanding of miRNA-regulated cancer pathways, including those specific miRNAs altered in HNSCC [8-10]. Recently, our miRNA profiles showed that *miR-133a* was down-regulated in cancer cells and that *miR-133a* had tumor suppressive functions [11-13]. Interestingly, *miR-1* and *miR-133a* are located on the same chromosomal locus, forming a so called cluster. *miR-1* and *miR-133a* are expressed in muscle and might be the most studied miRNAs in skeletal and cardiac muscle development [14,15].

*miR-1* was also down-regulated in our miRNA screening of hypopharyngeal and esophageal squamous cell carcinoma [13,16]. However, the functional significance of *miR-1* has not been clarified in HNSCC. The aim of this study was to investigate the function of *miR-1* in HNSCC cell lines and to identify *miR-1*-regulated cancer pathways. For target genes searches of *miR-1* in HNSCC cells, we performed genome-wide gene expression analysis. We focused on *transgelin 2* (*TAGLN2*) as a candidate target of *miR-1*, as it was among the most down-regulated genes. Insight into the association between tumor suppressive miRNA and their target oncogene networks could enhance our understanding of the molecular mechanism of HNSCC carcinogenesis.

## RESULTS

### Effect of miR-1 transfection on cell proliferation, migration, and invasion in HNSCC cell lines

**Figure 1:**
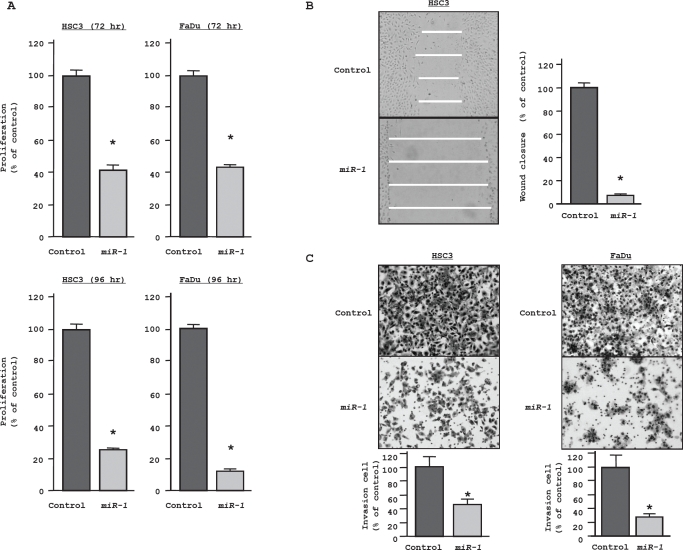
Gain-of-function studies using *miR-1* transfected HNSCC cell lines (HSC3 and FaDu) (A) Cell growth as revealed by the XTT assay after 72 hr [above] and 96 hr [below]. (B) HSC3 cell migration activity (wound healing assay).(C) Cell invasion activity (Matrigel invasion assay) in HSC3 and FaDu transfected with *miR-1*. *P < 0.05

To determine the function of *miR-1*, we performed gain-of-function analysis using *miR-1* transfectants. The XTT assay showed statistically significant inhibition of cell proliferation in *miR-1* transfectants in comparison with miRNA controls after 72 hr and 96 hr. For example, after 72 hr, both *miR-1* transfected HSC3 and FaDu cultures grew only ~42% as much as control cultures (both P < 0.05, Figure 1A, top), while after 96 hr, proliferation fell to 23% and 9% of controls, respectively, (both P < 0.05, Figure 1A, bottom). Wound healing assays of *miR-1-*transfected HSC3 demonstrated that cell migration was significantly inhibited to 5% that of control (P < 0.05; Figure 1B).The Matrigel invasion assay showed that the number of invading cells was significantly decreased in *miR-1* transfectants. Relative to control values (100%), the percentages of invading HSC3 and FaDu cells were only 45% and 27.1%, respectively (both P < 0.05; Figure 1C).

### Effect of miR-1 transfection on cell apoptosis and cell cycle in HNSCC cell lines

**Figure 2:**
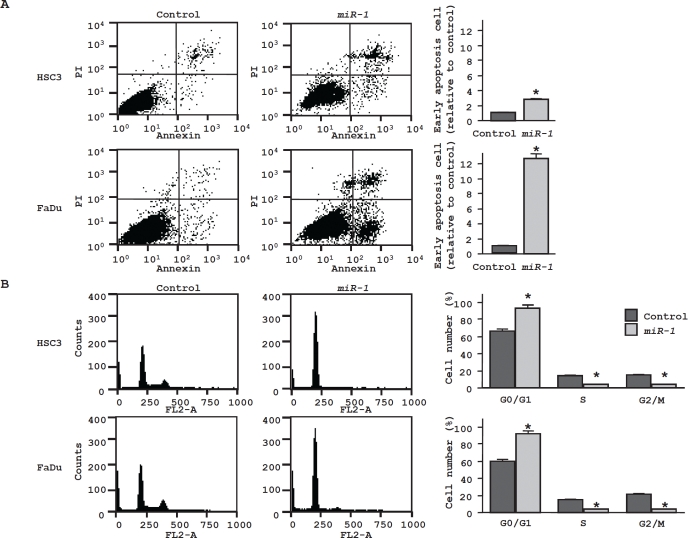
Effect of *miR-1* transfection on cell apoptosis and cell cycle (A) The representative quadrant figures of miRNA-control, or *miR-1* transfectants in HSC3 [above] and FaDu [below] cells are shown. The bar chart shown on the right of each quadrant indicates the ratio of the early apoptotic cell fraction in *miR-1* transfectants in comparison with miRNA-control transfectant. The data for the early apoptotic cell fraction is expressed as the relative value of the average expression of the miRNA-control transfectant. * P < 0.05. (B) The typical figures of cell cycle analysis of miRNA-control, or *miR-1* transfectants are shown. The bar chart shown on the right of each figures represents the percentage of the cells in G0/G1, S, or G2/M phase as indicated. * P < 0.05.

Cell apoptosis in *miR-1* transfected cells was assessed by flow cytometry. The fraction of early apoptotic cells significantly increased in *miR-1* transfectants approximately 3-fold in HSC3 and 12-fold in FaDu compared with controls (both P < 0.05, Figure 2A). We also confirmed induction of apoptosis approximately 4-fold in FaDu by ectopic *miR-1* performing TUNEL assay (data not shown). As for cell cycle distribution, cells in G0/G1 phase were significantly greater in *miR-1* transfectant than those in the control (Figure 2B). These results suggest that ectopic *miR-1* expression induces G0/G1 arrest in both HNSCC cell lines

### Gene expression profiling identifies down-regulated genes in miR-1 transfectants

To investigate candidate molecular targets of *miR-1* in HNSCC cells, we examined the effect of *miR-1* on protein coding genes. Mature *miR-1* was transiently transfected into HSC3 and FaDu cells, with negative miRNA transfection used as a control. Comprehensive gene expression analysis (see Methods) clearly showed changes in gene expression patterns between *miR-1* and negative-control transfectants. To identify candidate *miR-1* target genes, a cut-off of value less than -2.00-fold was applied to the array data. This filter resulted in the identification of 59 genes that were significantly down-regulated upon *miR-1* transfection in both HSC3 and FaDu cells (top 20 genes are shown in Table 1). Entries from the microarray data were approved by the Gene Expression Omnibus (GEO), and were assigned GEO accession number GSE24782. The 3' UTR of these down-regulated genes were examined for *miR-1* target sites using the TargetScan database. Of the top 20 putative gene targets, 17 genes contained *miR-1* target sites.

**Table 1:** Down-regulated genes in miR-1 transfectants

No.	Entrez Gene ID	Gene Symbol	Gene Name	Log2 ratio			*miR-1 target site*
				HSC3	FaDu	Average	
1	27230	SERP1	stress-associated endoplasmic reticulum protein 1	−1.61	−2.54	−2.08	+
2	8407	TAGLN2	transgelin 2	−1.77	−2.03	−1.90	+
3	23446	SLC44A1	solute carrier family 44, member 1	−1.62	−1.96	−1.79	+
4	9542	NRG2	neuregulin 2	−1.87	−1.60	−1.73	−
5	23531	MMD	monocyte to macrophage differentiation-associated	−1.38	−2.03	−1.71	+
6	5756	TWF1	twinfilin, actin-binding protein, homolog 1 (Drosophila)	−1.57	−1.69	−1.63	+
7	5819	PVRL2	poliovirus receptor-related 2 (herpesvirus entry mediator B)	−1.08	−2.02	−1.55	+
8	201895	C4orf34	chromosome 4 open reading frame 34	−1.53	−1.50	−1.52	+
9	3927	LASP1	LIM and SH3 protein 1	−1.33	−1.61	−1.47	+
10	7106	TSPAN4	tetraspanin 4	−1.26	−1.63	−1.45	+
11	4860	NP	nucleoside phosphorylase	−1.40	−1.48	−1.44	+
12	5757	PTMA	prothymosin, alpha	−1.33	−1.49	−1.41	+
13	84650	EBPL	emopamil binding protein-like	−1.48	−1.34	−1.41	+
14	303	ANXA2P1	annexin A2 pseudogene 1	−1.65	−1.15	−1.40	−
15	8683	SFRS9	splicing factor, arginine/serine-rich 9	−1.36	−1.38	−1.37	+
16	359845	FAM101B	family with sequence similarity 101, member B	−1.03	−1.68	−1.36	+
17	726	CAPN5	calpain 5	−1.46	−1.23	−1.35	+
18	64420	SUSD1	sushi domain containing 1	−1.25	−1.44	−1.34	+
19	9881	TRANK1	tetratricopeptide repeat and ankyrin repeat containing 1	−1.24	−1.40	−1.32	−
20	79794	C12orf49	chromosome 12 open reading frame 49	−0.98	−1.64	−1.31	+

### TAGLN2 is a target of post-transcriptional repression by miR-1

**Figure 3:**
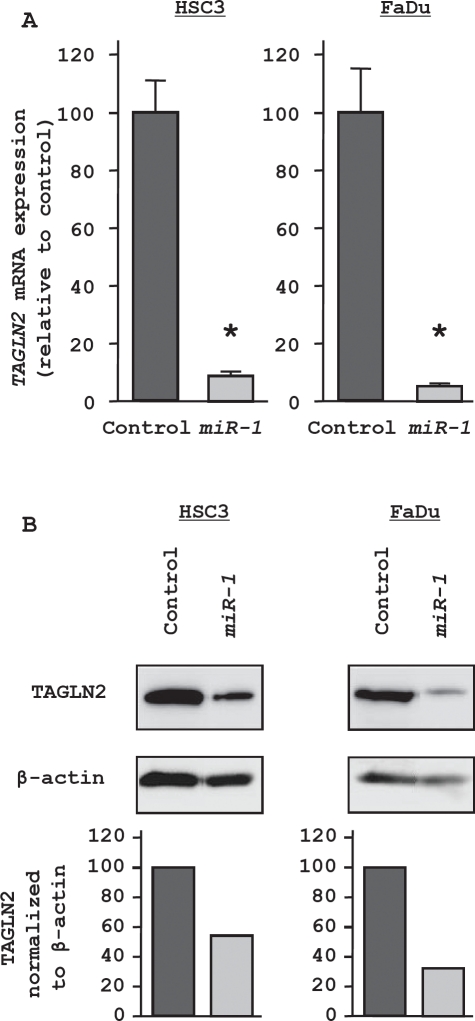
Regulation of *TAGLN2* expression in *miR-1* transfectants (A) RT-PCR revealed that *TAGLN2* mRNA was markedly repressed in *miR-1* transfectants compared with the miRNA-controls. * P < 0.05 (B) Western blots revealed that TAGLN2 protein was also decreased in *miR-1*.

**Figure 4:**
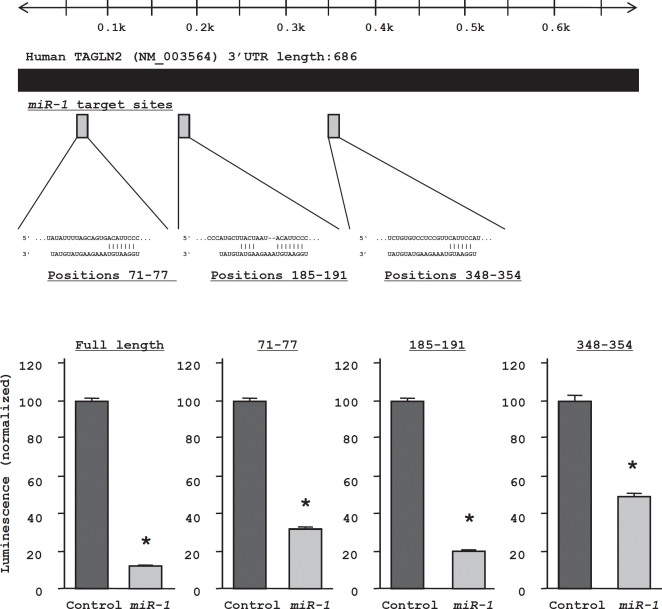
Three target sites for *miR-1* in the *TAGLN2* 3'UTR were identified with the TargetScan database A luciferase reporter assay using the vector encoding full-length 3'UTR and respective putative target sites of *TAGLN2* mRNA. The *Renilla* luciferase values were normalized by firefly luciferase values. * P < 0.05

*TAGLN2* was the second ranked candidate gene in the genome-wide gene expression analysis. We focused on *TAGLN2* and not the top ranked gene (*SERP1*), because the latter was not reported to be associated with carcinoma. The expression level of *TAGLN2* mRNA was significantly decreased in both HNSCC cell lines (HSC3 and FaDu) transfected with *miR-1* (Figure 3, upper). The protein expression levels were also markedly reduced in *miR-1* transfectants (Figure 3, lower). HSC3 cells were used to determine the mechanism of *miR-1* suppression of *TAGLN2* expression. The TargetScan database identified three putative target sites in the 3'UTR of *TAGLN2* (Figure 4, upper). A luciferase reporter assay confirmed the 3'UTR of *TAGLN2* as the actual target of *miR-1*. Luciferase activity was significantly decreased by the full length 3'UTR of *TAGLN2* as well as by all three predicted targeting sites of *miR-1* (positions 71-77, 185-191, and 348-354 in the 3'UTR of *TAGLN2*) (Figure 4, lower).

### Effects of TAGLN2 silencing on cell proliferation, apoptosis, migration, and invasion in HNSCC cell lines

**Figure 5:**
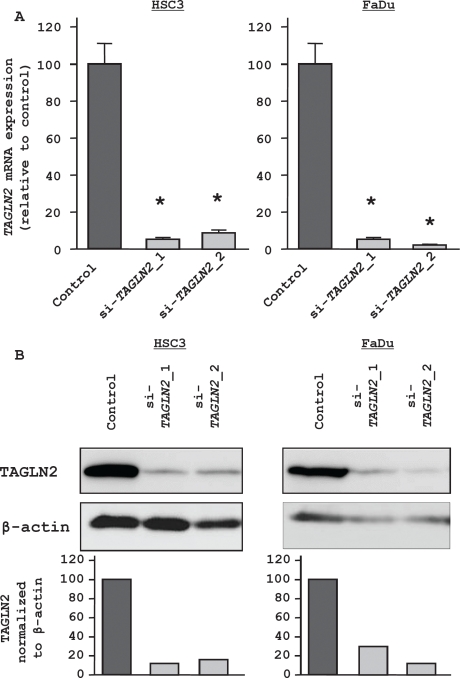
Effect of *TAGLN2* silencing on HNSCC cells (A) RT-PCR revealed that *TAGLN2* mRNA was markedly repressed in si-*TAGLN2* transfectants compared with the si-controls. * P < 0.0001 (B) Western blot revealed that TAGLN2 protein was also decreased in si-*TAGLN2*.

Loss-of-function assays using siRNA analysis were performed to examine the oncogenic function of *TAGLN2*. We asked whether si-*TAGLN2* reduced both mRNA and protein expression levels of si-*TAGLN2* transfectants in HSC3 and FaDu. Both *TAGLN2* mRNA and TAGLN2 protein were reduced following a 72 hr transfection with si-*TAGLN2* (Figure 5). The XTT assay revealed significant cell growth inhibition in si-*TAGLN2* transfected cells after 96 hr transfection. Specifically, with si-*TAGLN2*_1 and si-*TAGLN2*_2,HSC3 proliferation was inhibited

**Figure 6:**
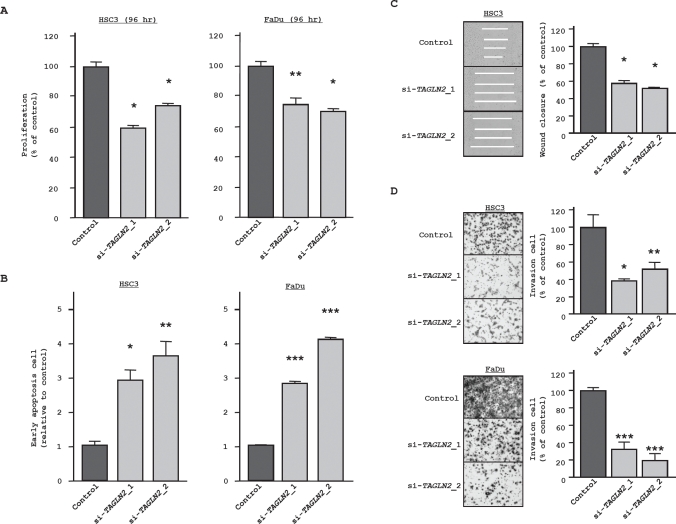
Loss-of-function studies using si-*TAGLN2* transfected HNSCC cell lines (A) Cell growth as revealed by the XTT assay. * P < 0.0001, ** P = 0.0003 (B) Apoptosis assay determined by flow cytometry. The apoptosis assay data are shown as normalized ratios in the histogram. * P = 0.0015, ** P = 0.002, *** P < 0.0001 (C) Cell migration activity (wound healing assay) in HSC3. * P < 0.0001; (D) Cell invasion activity (Matrigel invasion assay) transfected with si-*TAGLN2*. * P < 0.0035, ** P < 0.0153, *** P < 0.0001

60.0% and 76.3%, respectively while FaDu was inhibited 76.8% and 69.2%, respectively (Figure 6A). Significant inhibition was not demonstrated after 72 hr transfection (data not shown). The fraction of early apoptotic HSC3 cells was increased in the two si-*TAGLN2* transfectants from 1.00 in controls to 2.91 ± 0.32 (si-*TAGLN2*_1) and 3.76 ± 0.23 (si-*TAGLN2*_2)(Figure 6B). Similarly, with FaDu cells, apoptosis increased from 1.00 (control) to 2.83 ± 0.03 (si-*TAGLN2*_1) and 4.13 ± 0.04 (si-*TAGLN2*_2). The wound healing assay demonstrated that HSC3 cell migration was inhibited by 59.6 ± 3.2% (si-*TAGLN2*_1) and 54.9 ± 3.7 (si-*TAGLN2*_2) (Figure 6C). Finally, the Matrigel invasion assay showed that the number of invading cells was significantly decreased in si-*TAGLN2* HSC3 transfectants relative to controls: 41.1 ± 3.5% (si-*TAGLN2*_1) and 54.5 ± 14.5 (si-*TAGLN2*_2). Similarly, FaDu transfectants were inhibited 29.4 ± 2.2% (si-*TAGLN2*_1) and 21.8 ± 1.3%(si-*TAGLN2*_2) (Figure 6D).

### TAGLN2 mRNA and miR-1 expression in HNSCC clinical specimens

**Figure 7:**
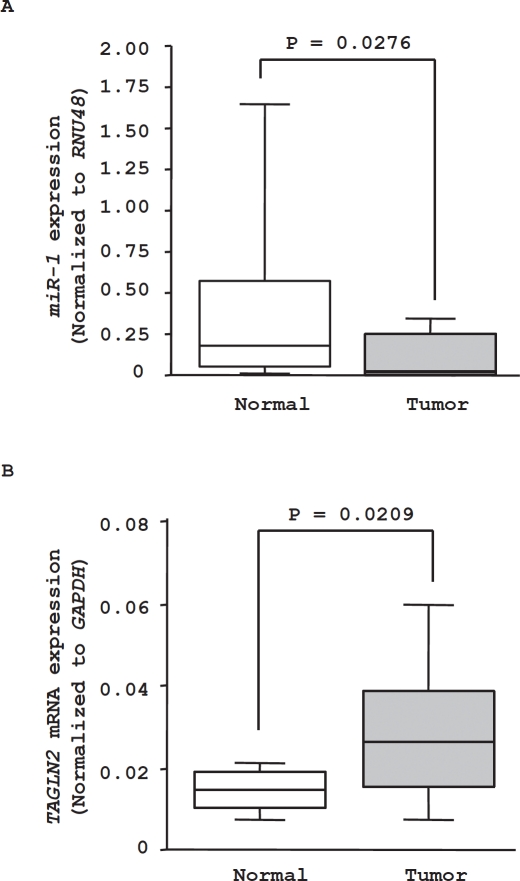
*miR-1* and *TAGLN2* mRNA expression levels in clinical specimens (A) *miR-1* expression in normal adjacent tissues and tumor tissues in 20 clinical specimens of HNSCC. (B) *TAGLN2* mRNA expression in tumor tissues and normal adjacent tissues in 20 clinical HNSCC specimens.

Data characterizing 20 HNSCC patients are presented in Table 2. The expression levels of *miR-1* were significantly down-regulated in clinical HNSCC specimen compared with adjacent normal tissues (P = 0.0276, Figure 7A). Conversely, *TAGLN2* mRNA was significantly up-regulated in tumor tissues (P = 0.0209, Figure 7B).

**Table 2:** Clinical features of HNSCC patients

No.	Gender	Age	Location	Differentiation	T	N	M	Stage
1	Male	82	Tongue	Well	1	0	0	I
2	Male	64	Tongue	Moderate	3	2b	0	IVA
3	Male	66	Tongue	Well	2	0	0	II
4	Male	70	Tongue	Well	1	0	0	I
5	Male	67	Oral floor	Moderate	3	2b	0	IVA
6	Male	47	Oral floor	Moderate	1	2a	0	IVA
7	Male	69	Larynx	Well	3	0	0	III
8	Male	73	Larynx	Well	2	0	0	II
9	Male	80	Larynx	Poor	3	2b	0	IVA
10	Male	59	Oropharynx	Poor	4a	0	0	IVA
11	Male	76	Oropharynx	Poor	2	0	0	II
12	Male	55	Oropharynx	Moderate	3	2c	0	IVA
13	Female	83	Oropharynx	Moderate	1	0	0	I
14	Male	74	Oropharynx	Well	2	0	0	II
15	Male	66	Hypopharynx	Moderate	2	2c	0	IVA
16	Male	71	Hypopharynx	Poor	2	2b	0	IVA
17	Male	49	Hypopharynx	Moderate	2	2b	0	IVA
18	Male	71	Hypopharynx	Poor	4a	2b	0	IVA
19	Male	66	Hypopharynx	Well	4a	2c	0	IVA
20	Male	66	Hypopharynx	Moderate	3	2c	0	IVA

## DISCUSSION

Aberrant expression of miRNAs and dysregulated gene expression of tumor suppressors and oncogenes have been associated with the development of human cancers. For the purpose of identification of cancer-related miRNAs, we determined miRNA expression signatures in hypopharyngeal and esophageal squamous cell carcinomas. Those profiles showed that *miR-1* was significantly down-regulated and had the features of a candidate tumor suppressor [13,16]. Other researchers have found that *miR-1* was down-regulated in other types of cancer[17-20]. Ectopic expression of *miR-1* inhibited cancer cells in hepatocellular carcinoma, rhabdomyosarcoma, and lung cancer[18-20].

Interestingly, *miR-1-1/miR-133a-2*, *miR-1-2*/*miR-133a-1*, and *miR-206*/*miR-133b* are clustered on three different chromosomal regions in the human genome, 20q13.33, 18q11.2, and 6p12.1, respectively. *miR-206* is similar to *miR-1* in terms of expression and function but differs from the *miR-1* sequence by four nucleotides. *miR-133a-1* and *miR-133a-2* possess identical mature sequences. *miR-133b* differs from *miR-133a* by a single nucleotide at the 3' end [15]. Regarding *miR-133a* in human cancers, our previous studies demonstrated that *miR-133a* functions as a tumor suppressor and targets multiple oncogenes, such as *FSCN1*[12,13,21], *LASP1* [22], *CAV1* [23] and *GSTP1* [24,25]. Other studies revealed that *miR-133a* was reduced in pancreatic ductal adenocarcinoma [26], colorectal carcinoma [27], tongue squamous cell carcinoma [28], and rhabdomyosarcoma [29]. It is clear that regulation of target genes by *miR-133a* is deeply involved in the modulation of cancer cells.

In this study, our data revealed that restoration of *miR-1* expression suppressed cell proliferation, migration, and invasion and promoted apoptosis and cell cycle arrest in HNSCC cells. Our results and those of past reports indicate that *miR-1* frequently functions as a tumor suppressor in human cancer.

miRNAs control the expression of target genes which contribute to cancer development and progression. Because it is important to identify novel miRNA-mediated cancer pathways, we investigated *miR-1*-regulated oncogenic targets. We adopted a method of genome-wide gene expression analysis in two HNSCC cell lines, using *miR-1* transfectants to identify targets. This strategy has led to the identification of tumor suppressive miRNAs targets [12,13,16,21,23,25]. Published articles revealed that *miR-1* mediates cell apoptosis, targeting *BCL2* in cardiac muscles [30]. In cancer, *miR-1* induced apoptosis through repression of *Mcl-1* in lung cancer [19]. *miR-1* also targets *c-Met* in rhabdomyosarcoma [20]. However, to our knowledge, the target gene of *miR-1* in HNSCC was unknown. *TAGLN2* was significantly down-regulated by ectopic expression of *miR-1* in HNSCC cell lines in our present study. The luciferase reporter assay revealed that *TAGLN2* contains three sites that actually bind *miR-1*. This is the first report demonstrating that tumor suppressive *miR-1* directly regulates *TAGLN2* in HNSCC cells.

TAGLN2 is a member of the calponin family of actin-binding proteins. TAGLN2 is a homolog of the protein TAGLN [31]. Though over-expression of TAGLN2 was observed in hepatocellular carcinoma, lung adenocarcinoma, and pancreatic cancer [32-35], analysis of TAGLN2 in HNSCC has not yet been reported. Here, we demonstrated significant up-regulation of *TAGLN2* expression in HNSCC clinical specimens. It was demonstrated that increasing *TAGLN2* expression was correlated with lymph node metastasis, distant metastasis, and the TNM classification in colorectal cancer [36]. Those results support the present loss-of-function analysis with si-*TAGLN2*, confirming that *TAGLN2* mediates cell migration and invasion, suggesting that this gene may have an oncogenic function. However, it is still unknown how TAGLN2, an actin-binding protein, contributes to cell apoptosis, which should be clarified by further analysis.

In conclusion, our results show that restoration of *miR-1* in cancer cells inhibits cell proliferation, invasion, and migration, supporting the hypothesis that *miR-1* functions as a tumor suppressor in HNSCC. Our data also indicate that *TAGLN2* may have an oncogenic function which is directly regulated by *miR-1*. The identification of novel *miR-1*-regulated cancer pathways could provide new insights into potential molecular mechanisms and applications to diagnosis, therapy, and prevention of the disease.

## METHODS

### HNSCC cell culture

Human HNSCC cell lines (HSC3, derived from a lymph node metastasis of tongue squamous cell carcinoma, and FaDu, derived from a primary lesion of hypopharyngeal squamous cell carcinoma) were provided by the American Type Culture Collection (ATCC, Manassas, VA, USA). Both cell lines were grown in Dulbecco's Modified Eagle's Medium/Nutrient Mixture F-12 Ham (DMEM/F-12) supplemented with 10% fetal bovine serum in a humidified atmosphere containing 5% CO2 at 37°C.

### RNA isolation

Total RNA was isolated using TRIzol reagent (Invitrogen, Carlsbad, CA, USA) according to the manufacturer's protocol. RNA concentrations were determined spectrophotometrically, and molecular integrity was checked by gel electrophoresis. RNA quality was confirmed using an Agilent 2100 Bioanalyzer (Agilent Technologies, Santa Clara, CA, USA).

### Mature miRNA transfection and small interfering RNA treatment

The following RNA species were used in this study: mature miRNAs, Pre-miR™ miRNA Precursors (hsa-miR-1; Pre-miR ID: PM10633), negative control miRNA (P/N: AM17111) (Applied Biosystems, Foster City, CA, USA), small interfering RNA (Stealth Select RNAi™ siRNA; si-TAGLN2 Cat#; HSS144745 and HSS144746) (Invitrogen) and negative control siRNA (D-001810-10; Thermo Fisher Scientific, Waltham, MA, USA). RNAs were incubated with Opti-MEM (Invitrogen) and Lipofectamine™ RNAiMax reagent (Invitrogen) as described previously [11]. Transfection efficiency of Pre-miR™ in cell lines was confirmed based on down-regulation of TWF1(PTK9) mRNA following transfection with miR-1 as previously reported [12,13,16].

### Cell proliferation, migration and invasion assays

Cells were transfected with 10 nM miRNA and siRNA by reverse transfection and plated in 96 well plates at 3 × 10^3^ cells per well. After 72 hr or 96 hr, cell proliferation was determined by the XTT assay, using the Cell Proliferation Kit II (Roche Molecular Biochemicals, Mannheim, Germany) [13,16]. Triplicate wells were measured for cell viability in each treatment group.

Cell migration activity was evaluated using a wound-healing assay. HSC3 was plated in six well plates at 2 × 10^5^ cells per well, and the cell monolayers were scraped using a micropipette tip. The initial gap length (0 hr) and the residual gap length (24 hr after wounding) were calculated from photomicrographs [12]. FaDu was not suitable for the wound healing assay because the cell monolayer tended to peel off during scraping.

A cell invasion assay was carried out using modified Boyden chambers containing transwell-precoated Matrigel membrane filter inserts with eight μm pores in 24 well tissue culture plates at 1 × 10^5^ cells per well (BD Biosciences, Bedford, MA, USA)[12]. Triplicate wells were measured for cell invasion in each treatment group.

### Flow cytometry

HSC3 and FaDu cells transiently transfected with miRNA-control, *miR-1*, siRNA-control and si-*TAGLN2* were harvested 72 hr after transfection by trypsinization. After the double staining with FITC-Annexin V and Propidium iodide (PI) was done using the FITC Annexin V Apoptosis Detection Kit (BD Biosciences) according to the manufacturer's recommendations, the cells were analyzed with a flow cytometry (FACScan®; BD Biosciences) equipped with a CellQuest software (BD Biosciences). Cells were discriminated into viable cells, dead cells, early apoptotic cells, and apoptotic cells, and then the relative ratio of early apoptotic cells to miRNA-control transfectant from each experiment were compared. Cells for cell cycle analysis were stained with PI using the CycleTEST™ PLUS DNA Reagent Kit (BD Biosciences) following the protocol and analyzed by FACScan. The percentage of the cells in G0/G1, S, and G2/M phase were counted and compared. Experiments were done in triplicate.

### Target gene search for miR-1

A genome-wide screen using *miR-1* transfectants was performed to identify target genes of *miR-1* in two HNSCC cell lines, HSC3 and FaDu. Oligo-microarray human 44K (Agilent Technologies) was used for expression profiling of the transfectants in comparison with a miRNA-negative-control transfectant [12,13,16]. Hybridization and wash steps were performed as previously described [37]. The arrays were scanned using a Packard GSI Lumonics ScanArray 4000 (Perkin Elmer, Boston, MA, USA). The data were analyzed by means of DNASIS array software (Hitachi Software Engineering, Tokyo, Japan), which converted the signal intensity for each spot into text format. The log2 ratios of the median-subtracted background intensities were analyzed. Data from each microarray study were normalized by a global normalization method [37].

Predicted target genes and their target miRNA binding site seed regions were investigated using TargetScan (release 5.1, http://www.targetscan.org/). The sequences of the predicted mature miRNAs were confirmed using miRBase (release 16.0, http://microrna.sanger.ac.uk/).

### Real-time quantitative RT-PCR

First-strand cDNA was synthesized from one μg of total RNA using a High Capacity cDNA Reverse Transcription Kit (Applied Biosystems). Gene-specific PCR products were assayed continuously using a 7900-HT Real-Time PCR System according to the manufacturer's protocol. The initial PCR step consisted of a ten min hold at 95°C, followed by 40 cycles consisting of a 15 sec denaturation at 95°C and a one min annealing/extension at 63°C. TaqMan® probes and primers for *TAGLN2* (P/N: Hs00761239_s1) and the *GAPDH* (A/N: NM_002046) internal control were obtained from Applied Biosystems (Assay-On-Demand Gene Expression Products). The expression levels of *miR-1* (P/N: PM10617) were analyzed by TaqMan quantitative real-time PCR (TaqMan® MicroRNA Assay; Applied Biosystems) and normalized to *RNU48* (A/N: X96648). All reactions were performed in triplicate, and included negative control reactions that lacked cDNA.

### Immunoblotting

Cells were harvested 72 hr after transfection and lysates were prepared. Fifty μg of protein lysate was separated by NuPAGE on 4 - 12% bis-tris gels (Invitrogen) and transferred to PVDF membranes. Immunoblotting was performed with diluted (1:150) polyclonal TAGLN2 antibody (HPA001925; Sigma-Aldrich, St. Louis, MO, USA), with β-actin antibody (sc-1615; Santa Cruz Biotechnology, Santa Cruz, CA, USA) used as an internal control. The membrane was washed and incubated with goat anti-mouse IgG (H+L)-HRP conjugate (Bio-Rad, Hercules, CA, USA). Specific complexes were visualized by echochemiluminescence (GE Healthcare Bio-Sciences, Princeton, NJ, USA), and the expression level of these genes was evaluated by ImageJ software (ver.1.43; http://rsbweb.nih.gov/ij/index.html).

### Plasmid construction and dual-luciferase assay

MiRNA target sequences were inserted between the XhoI–PmeI restriction sites in the 3'UTR of the hRluc gene in the psiCHECK™-2 vector (C8021; Promega, Madison, WI, USA). Primer sequences for full-length 3'UTR of *TAGLN2* mRNA (ATCGCTCGAGACAGATGGGCACCAACCGCG and CTCTAGGTTTAAACATCTTCCTCAAGCCCCAGAC) were designed. Specific miRNA target sequences (71–77: atattttagcagtgacattcccagagagccccaga gctct; 185-191: tcccccatgcttactaatacattcccttccccatagccat; 348-354: ctgagctctgtgtcctccgttcattccatggctgggagtc) for *miR-1* were artificially synthesized and inserted in the vector. Following that, HSC3 cells were transfected with 15 ng of vector, 10 nM of miRNA, and one μL of Lipofectamine™ 2000 (Invitrogen) in 100 μL of Opti-MEM™ (Invitrogen). The activities of firefly and *Renilla* luciferases in cell lysates were determined with a dual-luciferase assay system (E1910; Promega). Normalized data were calculated as quotients of *Renilla*/firefly Luciferase activities.

### Clinical HNSCC specimens

Twenty pairs of primary HNSCC (oral cavity, six; larynx, three; oropharynx, five; hypopharynx, six) and corresponding normal epithelial samples were obtained from patients in Chiba University Hospital (Chiba, Japan) from 2007 to 2009. All tissue specimens were obtained from patients undergoing surgical treatment. Normal tissues were obtained far from the center of the cancer in surgical specimens. No cancer cells were detected in neighboring formalin-fixed paraffin-embedded tissues. Written consent of tissue donation for research purposes was obtained from each patient before tissue collection. The protocol was approved by the Institutional Review Board of Chiba University. The specimens were immersed in RNAlater (Qiagen, Valencia, CA, USA) and stored at -20°C until RNA was extracted.

### Statistical analysis

The relationships between two groups and the numerical values obtained by real-time RT-PCR were analyzed using the nonparametric Mann-Whitney *U* test or the paired t-test. The relationship among three variables and numerical values was analyzed using the Bonferroni-adjusted Mann-Whitney U test; a non-adjusted statistical level of significance of P < 0.05 corresponded to a Bonferroni-adjusted level of P < 0.0167. All analyses were performed using Expert StatView (version 4, SAS Institute Inc., Cary, NC, USA).
